# Supplementary material for: Are carbon emissions trading and green financial instruments synergistic? -Comprehensive quantitative research based on content analysis
Source: PLoS One. 2024 Mar 7;19(3):e0298601. doi: 10.1371/journal.pone.0298601 (PMC10919601; doi:10.1371/journal.pone.0298601)
Supplement: S1 Data — (ZIP) [file pone.0298601.s001.zip › Data/地级市Z_F工作报告.zip!/║■─╧/│ñ╔│╩╨/└┤╫╘╣1⁄2╓┌║┼í╛┬φ┐╦╩2╛▌═°í┐_2014.docx]

各位代表：

　　现在，我代表市人民政府向大会报告工作，请予审议，并请各位政协委员和其他列席人员提出意见。

　　一、2013年工作回顾

　　2013年是新一届政府履职的第一年。在市委的坚强领导下，全市上下认真落实省委、省政府“四化两型”总战略和“三量齐升”总要求，大力开展“六个走在前列”大竞赛活动，科学应对复杂严峻的宏观经济形势，着力稳增长、调结构、转方式、保民生，完成了市第十四届人民代表大会第一次会议确定的目标任务。预计全市实现地区生产总值7200亿元，增长12%；固定资产投资4600亿元，增长20%；财政总收入883.89亿元，增长19.54%，其中公共财政预算收入536.63亿元，增长23.8%；规模工业增加值达2600亿元，增长14%；社会消费品零售总额2800亿元，增长14%；城乡居民收入增长12%左右；城镇登记失业率为2.89%；居民消费价格指数为102.8；全面完成节能减排任务。

　　（一）新型工业化和现代服务业融合发展。狠抓重大产业项目建设，着力构建多点支撑工业格局。上海大众、蓝思科技等项目顺利推进，基伍通讯、洋河酒业等项目成功签约，广告产业园、中联麓谷第二工业园开工建设。新增国家工程技术研究中心、重点实验室8家，中南大学科技园落户岳麓科技产业园，发明专利授权量居全国十强。汽车制造、电子信息等产业快速崛起，全部工业总产值突破9000亿元。长沙高新区、浏阳经开区和宁乡经开区、金霞经开区、望城经开区分别成为国家创新人才培养、再制造产业、新型工业化产业、知识产权示范（试点）基地（园区）。加强对内对外开放，促进服务业加快发展。新引进世界500强企业2家，中南商贸物流城、阿里巴巴长沙产业带签约落地，实现进出口总额98亿美元，增长12.5%。充分发挥外事侨务、对台、贸促会、口岸管理等重要作用。开展国家现代服务业综合试点，推进城市综合体建设和竣工运营，全市重点电子商务企业交易总额达520亿元，增长23.8%。推动金融生态区、滨江金融商务区建设，率先成立民间借贷服务中心。旅游收入突破千亿元大关。花明楼景区晋升5A级旅游景区。成功举办一批重要节会，推进国际会展中心建设，会展经济稳步发展。二三产业互动融合，有效促进了经济结构调整和发展方式转变。

　　（二）城市承载能力和整体形象显著提升。以规划引领城市发展，不断完善城市基础设施。城市总体规划修改成果上报国务院审批，开展片区控制性详规修改，启动中心城区排水等专项规划编制；实施“一江两岸、东提西拓”战略，提升先导区、高铁新城规划品质，完善湘江滨水区城市设计，省府新区、隆平新区等重点片区建设快速推进。地铁2号线试运行，长浏高速、湘府路大桥、南湖路湘江隧道、人民路东延线建成通车，地铁1号线、湘江综合枢纽、沪昆高铁长沙南站、河西综合交通枢纽、芙蓉北大道等重大项目有序推进，启动东、南二环改造，完成西二环改造。加强城乡环境建设，不断提升整体形象。大力开展市容市貌、城乡结合部环境、道路交通秩序、非法营运“四大整治”，拆除违法建设、户外广告招牌305万平方米。加强社区综合整治，有效激发社区组织活力。依法征拆补偿安置，推进棚户区和城中村改造。　　启动南郊公园改造，完成晓园公园景观提质，改建4条景观特色街，新建社区公园20个，新增城区绿地300公顷，森林覆盖率达53.48%。继续开展城乡环境整洁行动，推进10条城乡“绿道”工程建设。城市文明程度指数和未成年人思想道德建设工作测评均获全国省会和副省级城市第一，涌现出全国道德模范廖月娥等先进典型。长沙宜居宜游品质更加彰显，湖湘文化特色更加鲜明。

　　（三）新型城镇化与农业现代化协调发展。完善农村基础设施，改善农民生产生活条件。加快城乡一体化试点镇建设，新建或续建项目434个。完成小二型水库除险加固143座、山塘清淤5万亩，加强气象、水文服务，战胜前涝后旱等灾害，解决30万农村人口安全饮水问题；建设改造农村公路960公里；新建、改造10千伏线路380公里、低压线路1800公里；完成农村危房改造4461户；新建户用沼气池4484口；新建、续建乡镇污水处理厂12家；启动首个全国农村生态能源服务平台建设。发展特色优势产业，加快现代农业发展。乔口渔都、广联生猪等100个重大项目有序推进；农民专业合作社达4962家，农村土地流转总面积291万亩，粮食种植面积561.7万亩，新增万亩以上蔬菜示范片4个、专业蔬菜基地2.77万亩，创建畜禽标准化养殖场165个；粮食集团收购金健米业；农产品加工销售收入突破千亿元，增长23.4%；种植花卉苗木45万亩，建设集中连片油茶基地28个，发展林下经济50.5万亩；休闲农业实现经营收入105亿元。加强供销合作、移民工作。城乡统筹步伐加快，正朝着“农业高效、农村精美、农民富裕”的目标迈进。

　　（四）两型社会建设和配套改革深入推进。加快先导区发展，推进两型社会建设。洋湖湿地公园二期、桃花岭、梅岭公园开园，梅溪湖国际新城展示新形象。新引进和建设高端服务业项目总投资达600亿元。大围山森林公园获批国家生态旅游示范区，长沙县成为全省首个国家生态县，望城区获评全国科学发展特色县（区），长沙成为全球绿色城市、全国十佳生态文明建设示范城市。深化重点领域改革，推进节能减排财政政策综合示范。实施阶梯水价、电价、气价、生态补偿和排污权交易，开展集约节约用地考核评估和低效利用土地再开发试点，能源消费总量、单位GDP能耗稳步下降。推进第二轮环保三年行动计划，实施24小时空气质量数据动态发布和国控重点污染源在线监控，加强大气污染治理，淘汰黄标车3.1万辆；落实省政府湘江保护治理“一号重点工程”部署，全面实施流域治理和河道、砂场综合整治；建成坪塘、新港污水处理厂，启动主城区污水管网提质改造工程，主城区城市污水处理率达95.7%。试验区改革深入推进，两型发展成为长沙鲜明的标签和靓丽的名片。

　　（五）社会事业发展和民生保障富有成效。全面完成为民办实事工程，民生保障持续改善。新增创业主体约7万户，新增城镇就业14.9万人，城镇零就业家庭保持动态清零。工伤保险实现职业群体全覆盖，第九次调整企业退休人员养老金待遇。改扩建乡镇敬老院10所，城乡低保标准每人每月提高50元。完成公交企业整合重组，全面取消3元票价公交。实行新建居民住宅供水设施统建统管，改造城区居民住宅户表10万户。启动经适房上市，完成保障性住房和棚户区改造2.7万套，新增住房公积金缴存职工12万人。食品安全城市建设深入推进，食品检测合格率保持97%以上。加强市场物价预测监管，主要消费品价格基本平稳。加快社会事业发展，推进基本公共服务均等化。新改扩建城区中小学、公办幼儿园81所，改造义务教育薄弱学校197所，区县（市）全部通过义务教育均衡发展国家督导评估，获评全国终身教育先进城市。建成示范性文化站20个，完成中山亭提质改造，长沙国王陵遗址、胡耀邦故居晋升全国重点文物保护单位，成为首批国家公共文化服务体系示范区。推进县级医院试点改革，市公共卫生中心、口腔医院南院投入使用，基层卫生服务能力不断提升。成功举办市第八届运动会，群众体育设施实现社区、行政村全覆盖。加强和创新社会管理，共建共享社会和谐。全面建成“天网工程”，保持对犯罪活动的高压态势，荣获全国社会管理综合治理“长安杯”。加强重大矛盾纠纷人民调解和法律援助，信访秩序明显好转。加强安全生产监管，推进企业安全生产标准化建设，安全生产形势总体稳定。健全人口网格化信息管理系统和计划生育利益导向政策体系，人口自然增长率为5.23％。。夯实统计基层基础，第三次经济普查顺利推进。加强国家安全、应急管理、防震减灾、民族宗教、档案、市志等工作。国防、人防和后备力量建设不断加强，在抗险救灾和促进稳定中发挥了重要作用，军政军民团结进一步巩固。各民主党派、工商联及工会、共青团、妇联、科协、文联、侨联、社科联、残联、红十字会、慈善会、关工委、老龄委等为长沙和谐发展作出新贡献。

　　（六）政府作风建设和执行能力不断加强。坚持依法科学决策，深入推进政务公开。全面开展行政决策法律审查，完善政府合同审查管理机制。认真落实人大及其常委会的决议决定，主动接受人大和政协监督，积极听取民主党派、工商联和无党派人士意见，全面完成人大建议和政协提案办理。做好省级部分管理权限下放的承接工作。办好政府门户网站，开通“12345”市民服务热线，实行非行政许可和年检项目网上办理。坚持精细管理，加强政府廉政建设。加强公共项目资金和国资监管，推行全过程预算绩效管理，财政支出效益明显提升，国资监管实现全覆盖。开展政府性债务和财政专项资金审计，完成政府投资审计项目878个，核减投资额28.6亿元，综合核减率17.95%。深入开展行政效能监察，坚决纠正部门和行业不正之风。坚持勤俭节约，机构编制和机关事务管理不断规范，专项治理超编车、超标车，全面清理办公用房，压缩“三公”经费支出，公务出国人次零增长，干部作风明显转变。

　　各位代表！这些成绩的取得，是省委、省政府和市委正确领导的结果，是历届市委、市人民政府打下坚实基础的结果，是人大、政协监督支持的结果，是全市上下共同努力的结果。在此，我代表市人民政府向全市各族人民，向各民主党派、工商联、人民团体和各界人士，向驻长部队、武警官兵和政法干警，向所有关心支持长沙发展的朋友们，表示诚挚的感谢和崇高的敬意！

　　回顾过去一年，我们在困难复杂的宏观环境下，实现了稳中有进、稳中向好，成绩来之不易。我们深深地体会到：必须抢抓发展机遇。积极争取长沙发展进入国家战略，两型社会、节能减排、现代服务业成为国家试点，为未来发展赢得竞争优势。必须注重改革创新。以改革破解发展瓶颈，以创新增强发展活力，推动又好又快、率先发展。必须强化项目支撑。千方百计引进项目，攻坚克难推进项目，实施一批好项目、新项目，为可持续发展奠定坚实基础。必须坚持民生优先。将更多财力用于保障和改善民生，让群众更多更公平地分享改革发展成果。

　　在总结成绩的同时，我们也清醒地看到发展中存在不少的困难和问题：产业结构还没有得到根本性调整，转型创新发展任务依然繁重；城市规划、建设、管理还存在薄弱环节，“四大整治”还需进一步加强；雾霾天气对群众生活造成一定影响，环境治理亟待加大力度；社会保障能力和公共服务水平与群众期盼还有差距；政府服务和公务员队伍作风有待进一步改进。对此，我们高度重视，将采取切实有效措施加以解决。

　　二、2014年目标任务

　　2014年是落实党的十八届三中全会精神、全面深化改革的开局之年，也是落实市委十二届六次全会精神、实施“十二五”规划的关键之年。中央经济工作会议对今年的形势进行了全面分析，世界经济仍将延续缓慢复苏态势，不稳定不确定因素依然存在，新的增长动力尚不明朗，我国经济存在下行压力，但经济发展长期向好的基本面没有变，中央继续实施积极的财政政策和稳健的货币政策，经济社会发展仍处于大有可为的战略机遇期，我们完全有信心抓住机遇，保持经济合理增长，推进转型创新发展。

　　我们的信心源自全面深化改革的强劲动力。在我国改革开放新的重要关头，党的十八届三中全会提出了“完善和发展中国特色社会主义制度，推进国家治理体系和治理能力现代化”的总目标，吹响了全面深化改革的号角。中央对湖南提出了“东部沿海地区和中西部地区过渡带、长江开放经济带和沿海开放经济带结合部”的新定位，这是湖南的区位优势，也是长沙发展的重大机遇。长沙肩负着多项重点改革使命，只要我们发扬“心忧天下、敢为人先”的“长沙精神”，只争朝夕地深化改革，就一定能激发内在的强大潜力，分享更多的改革红利。

　　我们的信心源自“六个走在前列”的历史重托。省委、省政府明确要求长沙在加快转型创新发展、城市规划建设管理、两型社会建设、共建共享全面小康、维护社会和谐稳定、提高党的建设科学化水平中走在全省乃至全国前列，成为“一带一部”核心区、深化改革先行区、自主创新示范区、两型建设先导区，赋予更大的权限，给予更多的支持。有省委、省政府作为坚强后盾，有全省人民对省会建设的大力支持，只要我们解放思想、勇于担当，干在实处、走在前列，就能从追赶型发展走向引领型发展，在百舸争流的城市竞争中抢占先机、赢得主动、实现赶超。

　　我们的信心源自全市人民群众的美好愿景。市委十二届六次全会着眼于满足人民群众过上更好生活的新期盼，对长沙今后一段时期的发展进行了全面部署，明确提出率先建成“全面小康之市、两型引领之市、秀美幸福之市”，强力实施“产业倍增、收入倍加、城乡品质倍升”，加快现代化进程，为我们描绘了未来美好蓝图，激发了干事创业的热情。只要我们紧紧团结和依靠全市人民，用科学的理念推动发展，用勤劳的双手创造财富，用坚实的脚步追逐梦想，就能将美好愿景变为现实。

　　为贯彻落实中央，省委、省政府和市委的精神，2014年政府工作的总体要求是：深入贯彻落实党的十八大、十八届三中全会和中央、省委、市委经济工作会议精神，按照市委十二届六次全会的战略部署，坚持稳中求进、改革创新，以转变发展方式为主线，以全面深化改革为动力，以转型创新发展为目标，着力统筹城乡、改善民生、创新治理、转变作风，在全面推进“六个走在前列”、率先建成“三市”、强力实施“三倍”、加快现代化进程中，开创更好更快发展新局面。

　　综合考虑各种因素，今年经济社会发展的主要预期目标是：地区生产总值增长11.5%；财政总收入增长15%，公共财政预算收入增长13%；固定资产投资增长18%；规模工业增加值增长13%；社会消费品零售总额增长14%左右；单位地区生产总值综合能耗下降3%，化学需氧量、二氧化硫、氨氮、氮氧化物等减排指标完成省下达任务；城乡居民人均可支配收入分别增长12%；城镇登记失业率控制在4%以内；人口自然增长率在7.5％。以内；居民消费价格指数控制在103.5左右。

　　实现上述目标，必须全面深化改革，坚持稳中求进、新中求进、好中求进，进一步解放思想、解放和发展生产力、解放和增强社会活力，以改革稳增长、以改革调结构、以改革惠民生，在工作中牢牢把握以下几点：

　　一是更加注重市场作用。使市场在资源配置中起决定性作用，大幅度减少政府对资源的直接配置，能由市场配置的资源逐步交由市场配置，能由市场提供的公共服务逐步实现政府购买；市场能有效调节的经济活动一律取消行政审批，企业投资项目一律由企业依法自主决策，依靠市场力量推动更好更快发展。

　　二是更加注重质量效益。推动经济发展由注重量的增长向更加注重质的提升转变，由注重增量的扩张向更加注重存量的升级转变。以资源环境约束倒逼发展方式转变，使经济发展更多依靠科技进步、劳动者素质提高和管理创新驱动；以质量的提升促进效益提升，增加企业利润和财政收入，增加劳动所得和居民收入。

　　三是更加注重两型引领。自觉肩负起试验区的历史使命，积极探索两型社会建设的科学路径，将两型理念贯穿于长沙改革、发展、建设全过程。加强生态文明建设，经济发展和城乡建设更加注重环境保护，构建两型产业体系和两型体制机制，促进资源集约节约利用，当好全国两型社会建设的排头兵和先行者。

　　四是更加注重城乡一体。积极推进以人为核心的城镇化，全面放开县城和建制镇落户限制，促进农业转移人口有序实现市民化。优化城镇布局，依托现有山水脉络等独特风光，让城市融入大自然。推动城市和小城镇协调发展，促进产业与城镇融合发展，实现以城带乡、城乡一体，提高城镇化质量。

　　五是更加注重群众感受。把群众需要作为工作导向，高度重视社情民意，完善社会参与机制，尊重人民群众首创精神，充分调动基层的积极性、主动性。更加关心贫困群众和弱势群体的生活，妥善解决群众最关心、最直接、最现实的利益问题，更好地保障和改善民生，让改革发展的成果更多更公平地惠及全市人民。

　　三、2014年工作重点

　　着重抓好以下七个方面的工作：

　　（一）突出转型升级，推进新型工业化。提高工业发展质量。坚持科技创新驱动，着力引进一批有创新成果、有知识产权的创新团队和领军人才，支持企业组建研发平台和创新联盟，加强知识产权保护和运用，加快工业化与信息化融合，提升自主创新能力。巩固发展工程机械、食品等已有千亿产业，加快汽车及零部件、电子信息、两型住宅、新材料、生物医药等成长型产业发展，着力培育新的千亿产业。改造提升烟花、服装等传统产业，淘汰落后产能。注重产业配套，拉长优势主导产业链。实施重大项目带动。紧盯国内外大型品牌企业，着力引进一批技术含量高、带动能力强的新项目，切实提高履约率、开工率、投产率。加快上海大众、菲亚特二期、比亚迪汽车城、格力电器、蓝思科技三期、介面光电二期、基伍通讯、中联环境产业园等重大项目建设，尽快形成现实生产力。促进园区错位发展。优化园区功能定位，规范产业落户政策，促进产业集聚发展。开展集群式项目满园扩园行动，实施两型化管理提标提档行动，鼓励发展工业地产，打造现代城市工业经济综合体，提升园区档次和功能。规范乡镇工业发展，打造特色工业小区。支持高新区创建国家创新型科技园区、长沙经开区创建国家知识产权示范园区、雨花经开区创建国家低碳工业园区，加快将宁乡经开区、浏阳经开区培育成新的“千亿园区”，推动望城经开区、金霞经开区晋升国家级经济开发区，支持区校共建中南大学科技园、湖南大学科技园。

　　（二）突出发展服务业，提升竞争优势。发展新型服务业态。大力发展金融、文化创意、信息软件、科技研发等现代服务业，重点发展总部经济、后援中心、工业设计等新兴业态，加快建立以服务经济为主体的现代产业体系。加快移动电子商务基地建设，大力支持仓储物流发展，引进大型快递物流企业，促进电子商务加速壮大，支持雨花区争创国家电子商务示范基地。支持一批文化产业龙头企业发展，加快广告产业园、湘台文化创意产业园、后湖国际艺术区建设，着力打造文化创意中心，推进国际文化名城建设。打造中部消费中心。深入推进国家现代服务业综合试点，科学布局现代服务业集聚区，规划引导地铁沿线商业发展，加快中心城区批发市场有序外迁和提质改造，推动高岭商贸市场群、长株潭商圈发展，加快大河西和马王堆农产品物流中心、湾田国际、金桥国际商贸城等项目建设，推进台商总部大厦、保利西海岸等城市综合体建设，抓好芙蓉中央商务区、金融生态区、滨江金融商务区建设，规范民间借贷机构服务管理。加快国际会展中心建设，支持会展经济发展。引导高档餐饮酒店开发面向大众的消费，挖掘消费潜能。加强旅游与文化、生态融合发展，推进大围山、铜官窑、灰汤温泉等旅游区建设，繁荣歌厅酒吧文化，打响“快乐长沙、宜游胜地”品牌。推动对外经贸合作。引进开放型产业项目，打造一批优质的外贸商品基地，扶持一批进出口总额在1亿美元以上的行业龙头企业，进出口总额增长8%。争取跨境电子商务应用、文化创意自由贸易区等国家试点。增开国际航线。加快金霞保税物流中心、临空综合保税区建设，打造国家级转型示范出口基地。启动霞凝铁路口岸作业区建设，申报海关特殊监管区，建设电子口岸平台。鼓励本土企业走出去参与国际国内竞争合作，加强与泛珠三角、长三角、长江中游城市群经济协作，促进国际友好城市合作交流。

　　（三）突出建管并重，打造品质城市。坚持规划先导。规划中融入历史文化元素，尊重群众行为习惯，注重细节设计，体现人文关怀。采取多中心、组团式发展，适时启动行政区划调整，优化城市发展格局。注重加强重点地段、重点区域的城市设计，加快编制解放垸规划，整体规划开发月亮岛等湘江洲岛，加强湘江两岸建设项目景观审查，打造和谐美观、特色鲜明的城市天际线。有序推进滨水新区、省府新区、南湖片区、空港城、苏圫垸生态新城建设，提升高铁新城开发建设品质。完善功能配套。启动地铁3、4号线、高铁南站至黄花机场中低速磁悬浮轨道交通建设，加快地铁1号线、城际铁路建设，实现地铁2号线试运营。启动长株潭环线高速建设，加快长韶娄高速、京港澳高速辅道及星沙联接线建设。启动桐梓坡至鸭子铺通道建设、万家丽路快速化改造，推进营盘路和劳动路东延、车站北路和东二环北延，加快黄江公路、宁梅大道、金阳大道、滨河大道建设，完成东、南二环改造，打通红旗路等断头路。实施公交优先战略，全面推进公交都市建设。完成湘江综合枢纽主体建设，加快新港三期建设，提质改造湘江风光带，打造“百里滨水走廊”。实施城区干道架空线缆入地，加强城区防洪排渍、消防设施建设，改造城区地下管网和排水堵点。加强城市管理。合理安排建设时序，有效减少道路开挖。加强公园广场、出入城口及机场车站周边环境整治，严格治理城乡违法建设。推进老旧住宅区、背街小巷改造，启动雅塘片区、望城坡等区域提质改造。启动城区部分主干道立面改造，提升“一江两岸”主要景观轴线及周边亮化效果。推行网格化、精准化、系统化管理，既以人为本又严格执法，做到宣传、教育、执法有机结合，巩固“四大整治”成果，打造更加整洁有序、清新靓丽的省会形象。

　　（四）突出城乡一体，推进新型城镇化。加强农村基础设施建设。整合涉农资金，吸引社会投资，建立多元化可持续的资金保障机制，实行整体规划、集中投入、片区实施。启动农村公路提质改造三年行动，加大危桥改造力度，新建农村公路600公里，提质改造300公里。推进农村电网扩容改造，有序推进通讯光纤向行政村延伸。加强农田水利基本建设，完成重点小二型水库除险加固45座，骨干山塘清淤扩容3万亩，疏浚沟渠1000公里，解决30万农村人口饮水安全问题。新建乡镇污水处理厂10家，建成户用沼气池3000口。积极发展现代农业。加快建设现代农业综合配套改革试验区，培育现代农庄、专业合作社等新型生产经营主体，大力发展家庭农场。加强种业科技创新，稳定粮食生产，保障粮食安全。大力发展农产品精深加工，打造一批知名龙头企业和名优品牌。完善现代农产品物流网络，加大农产品直销配送。加强农业新技术、新品种和农业机械的推广应用，完善农业保险制度，促进农业增效、农民增收。推进重点城镇建设。按照生产空间集约高效、生活空间宜居适度、生态空间山清水秀的要求，积极稳妥推进以人为核心的城镇化。坚持慎砍树、不填湖、少拆房，尽可能保留村庄原始风貌，合理引导村民集中居住，融入现代元素改善居住条件。深入实施城乡环境整洁行动，巩固农村连片整治成果。有序推进15个重点小城市、中心镇、特色镇建设，试点探索社会发展总体规划、土地利用规划、城市建设规划、产业发展规划等“多规合一”，打造一批具有历史记忆和湖湘特色的美丽城镇，方便农村人口就近转移，辐射带动周边地区发展。

　　（五）突出两型建设，加快引领发展。发挥先导区示范作用。全面提升先导区规划、建设品质，大力发展两型交通，创建全国生态文明示范区。加快梅溪湖国际新城、大王山旅游度假区、洋湖总部经济区、滨江新城建设，提质改造滨江风光带等景观工程，开工建设市儿童公园、儿童医院、科技展示中心，完成洋湖湿地公园、巴溪洲公园建设。鼓励发展新能源和绿色建筑产业，大力发展循环经济产业，积极引进高端服务业，加快构建两型产业体系。加强生态环境保护。坚持“不破坏、少影响”原则，划定禁止开发、限制开发区域。加强环境风险企业管理，落实生态补偿制度。完成第二轮环保三年行动计划，加强年嘉湖、梅溪湖、松雅湖等水质保护。实施三年造绿大行动，加快城区主干道绿化提质，启动沙湾公园、八方山公园建设，新建社区公园20个，新增城区绿地600公顷；加快浏阳河生态经济示范区建设，创建绿色示范集镇10个、绿色示范村庄和社区各30个。大力治理环境污染。推行环境污染责任保险与年审制度，实行环境污染第三方治理和损害赔偿。强力推进省政府“一号重点工程”，加强湘江流域治理，实现湘江、浏阳河城区段全截污、全治理。加强扬尘源头治理，规范渣土运输和建筑工地现场管理；严格控制油烟、汽车尾气等污染源，引导新能源汽车发展，淘汰黄标车1万辆，确保雾霾天气明显减少。完成10万平方米畜禽养殖污染治理，妥善处置固体废弃物。推进能源资源节约。推广集约节约用地模式，规范工业用地管理，推进低效利用土地再开发。加速园区和重点新区分布式能源建设，着力推进可再生能源利用，推广建设工业园区厂房屋顶光伏电站，有效降低能源资源消耗。鼓励发展再制造产业。加大建筑工业化支持力度，新建大型公共建筑全部达到绿色建筑标准。

　　（六）突出公共服务，发展社会事业。提高教育质量。注重教育公平，加快学校标准化建设，不断改善办学条件，新改扩建城区中小学10所、公办幼儿园45所，着力缓解大班额、择校问题，提质改造义务教育薄弱学校130所。加强校长任职交流和教师对口支援，推进中招中考制度改革，完善综合素质评价，促进学生成长、教师进步。支持长沙学院发展。以商贸职院为主体整合相关资源筹办第二所本科院校。拓宽终身学习通道，创建全国学习型城市。繁荣文化事业。启动公共文化服务示范区提质提效三年行动计划，建设一批集中居住区文化广场，提质乡镇综合文化站30家，创建社区（村）示范文体活动中心100个，加强青少年活动基地和公共阅读场所建设，创作一批文化艺术精品。加强历史文化街区保护，加快长沙国王陵国家考古遗址公园、炭河里遗址公园建设。加强文化市场综合执法，规范文化市场秩序。鼓励非公有制文化企业发展，妥善解决市属文艺院团改革遗留问题。健全诚信体系，弘扬雷锋精神，推动志愿者服务制度化，促进文明创建常态化，引导市民自觉养成文明习惯。建设健康城市。注重人才引进和设施投入，推进市三医院、第二批县级公立医院综合改革试点。提质改造乡镇卫生院和社区卫生服务中心10所、村卫生室260所，增强农村医疗卫生服务能力。扩大基本药物目录范围，严格控制门诊、住院次均费用增幅，支持民营医院发展，切实减轻群众就医负担。构建全民健身公共服务体系，完善群众体育健身设施，积极备战省十二运会。加强计生基层基础工作，提升计生管理服务水平。理顺食品药品监督管理体制，严格食品药品监管执法，切实保障群众饮食和用药安全，积极创建全国食品安全示范城市。

　　（七）突出改善民生，促进社会和谐。推进创业富民。充分发挥非公有制经济在扩大就业中的主渠道作用，鼓励自主创业、二次创业，以创业带动就业。健全公共就业服务体系，加强技能培训和指导，开展劳动力转移培训，实施就业援助，抓好高校毕业生等重点群体就业。创建充分就业社区，城镇零就业家庭保持动态清零，促进更高质量的就业。加强社会保障。提高社会保险统筹层次，完善被征地农民社会保障制度，扩大农村低保、医保和养老保险覆盖面，提高赔付标准。改革医保支付制度，建立低收入家庭大病救助机制，完善居民医保门诊统筹。加快公共租赁住房和廉租住房建设，加大住房公积金在非公企业建制力度，推进黄兴北路二期、火车北站二期、燕山街、南湖片区等棚户区和国有工矿棚户区改造，创新零星棚户区改造模式，完成棚户区改造13260户。推进红星村、戴家河村、天马村等城中村改造，改造农村危房4200户。积极发展养老事业，鼓励发展社会化养老机构，启动乡镇敬老院建设三年行动计划，提升居家养老服务水平。发展慈善事业，加快市儿童福利院和乡镇社会救助站建设，建好市残疾人康复机构。实施价格惠民，推进住宅小区电力专变改公变，完成城区自来水户表改造20万户，公交票价优惠30%。创新社会治理。深化“天网工程”拓展应用，妥善处置各类突发事件和舆情，依法惩治各类违法犯罪，加强特殊人群管理，增强群众安全感。创新普法教育形式，提升全社会法律素养。实行网上受理信访，落实涉法涉诉信访依法终结机制，加强人民调解、法律援助，及时就地解决群众合理诉求。加强安全保障能力建设，突出隐患排查和专项整治，严防各类安全事故发生。加强社会组织扶持监管，加大社区（村）设施建设力度，完成第九次村委会换届选举。完成第三次全国经济普查，提高统计服务决策水平。促进民族团结，加强宗教场所建设。加强档案、市志、机关事务管理。保障妇女、儿童、老年人、残疾人合法权益。切实加强国防动员、人民防空和国家安全工作，推进国防教育基地建设，深化民兵预备役体制改革，促进军民融合深度发展。

　　四、切实加强和改进政府工作

　　贯彻落实十八届三中全会精神，使市场在资源配置中起决定性作用和更好发挥政府作用，全面完成今年目标任务，必须切实加强和改进政府工作。

　　坚持改革创新。当前经济社会发展受到的制约越来越多，要实现约束条件下的发展，唯一办法就是改革创新。更加解放思想。以解放思想推动观念更新，以解放思想推进全面深化改革，以解放思想引领转型创新发展。把解放思想与实事求是统一起来，坚持“五位一体”的发展理念，不简单地以GDP论英雄，同时立足社会主义初级阶段的基本国情，坚持以加快发展解决发展中的问题。转变政府职能。进一步理顺政府与市场、政府与社会的关系，统筹抓好政府机构改革和职能调整，理顺市与区县（市）、部门与部门关系，有效解决职能交叉问题。推动事业单位分类改革，推进政事分开。做好第一轮国企改革扫尾工作，引导国有资本、集体资本、非公有资本交叉持股，发展混合所有制经济，推进政企分开。推进简政放权。用好、用足、用活已承接的省级管理权限，全面清理市本级管理权限，凡是由基层管理更方便更有效的经济社会事项，一律下放至区县（市）、园区。勇于自我革命，减少审批环节，优化审批流程，确保行政审批效率大幅提升。率先实施商事登记制度改革，实现“先证后照”向“先照后证”转变，更好地调动社会投资创业的热情。

　　推进依法行政。依法行政是建设法治政府的核心，是政府工作的基本要求，要把依法行政贯穿于政府工作的全过程。依法行使职权。严格按照法定权限和程序行使权力、履行职责。落实政府法制工作规定，实行重大决策合法性审查。加强政府合同审查，保障政府和企业的合法权益。严格恪守诚信。坚决维护政策的稳定性和连续性，做到言必行、行必果，杜绝朝令夕改，以政府诚信带动社会诚信。树立正确的政绩观，不弄虚做假，不虚报浮夸，以没有水分、可持续的增长取信于民。政府工作人员要坚持职业操守，对人民群众承诺的事情要有诺必行、负责到底。主动接受监督。主动接受人大法律监督、工作监督，认真执行人大及其常委会的各项决定、决议。主动接受政协民主监督，积极开展民主协商。及时办理人大代表议案、建议和政协提案。高度重视民主党派监督、社会监督和舆论监督，自觉接受人民群众监督。

　　弘扬实干精神。实现全年目标任务，关键在于抓好落实。要把抓落实作为政府工作的生命线，作为检验干部作风的试金石。强化问题导向。从制约长远发展的重大问题、群众反映的难点热点问题入手，一项一项研究解决方案，制定详细周密的计划，加强执行的过程控制和结果评价，既要评价工作效率，也要评价综合效益，总结推广普遍性的经验，提高整体工作水平。强化时间意识。既要明确路线图，又要制定时间表，把握时间节奏，以目标倒逼进度、以督查倒逼落实，使各项工作始终处于一种高速运转、高效运行的状态，以高效率赢得高效益、以快节奏换来快发展。强化危机意识。居安思危，沉下心来研究工作，善于发现苗头性、倾向性的问题，未雨绸缪，提前应对。既要纵向比，更要横向比，更多地正视自身不足和差距，增强加快发展的紧迫感。强化进取意识。树立争先进位的勇气和信心，坚决摒弃骄傲自满、停滞不前的思维定势，不回避矛盾，不畏惧困难，甘于奉献，勇于担当，创造性地推动工作落实。

　　加强廉政建设。廉洁是政府公信力的基石。要对人民、对法律、对权力始终抱有敬畏心，管住权力、管好钱财，清廉从政、勤俭为民。坚决反对“四风”。深入开展党的群众路线教育实践活动，深入基层、深入企业、深入群众，倾听群众呼声，集中群众智慧，着力解决群众反映强烈的突出问题。坚持勤俭办一切事业，严格控制“三公”经费支出，坚决反对和根治形式主义、官僚主义、享乐主义、奢靡之风。加强制度建设。用制度管权管钱管人，健全严格的财务预算、核准和审计制度，管好财政资金、公共资产。继续推进全口径预算管理，筑牢预算约束力防线，防范政府债务风险。落实党风廉政建设责任制，强化廉政教育基础作用和领导干部监督，科学有效预防腐败，坚决惩治腐败，把权力关进制度的笼子里。深化政务公开。突出公共财政、价格收费、资源交易等重点领域，及时主动公开涉及群众切身利益的公共信息，做好公民依申请公开，探索网络时代政务公开的新形式，完善基层政务公开平台，提升市政务中心服务功能，让权力公开透明运行，以廉政赢得人民群众的信赖。

　　各位代表，长沙的发展站在了新的起点，新形势、新任务催人奋进。让我们在市委的正确领导下，按照“六个走在前列”的要求，求真务实，改革创新，为率先建成“三市”、强力实施“三倍”、加快现代化进程而努力奋斗！
